# Research advances in treatment methods and drug development for rare diseases

**DOI:** 10.3389/fphar.2022.971541

**Published:** 2022-10-12

**Authors:** Qiaoqiao Han, Hengtao Fu, Xiaoyue Chu, Ruixin Wen, Miao Zhang, Tao You, Peng Fu, Jian Qin, Tao Cui

**Affiliations:** ^1^ School of Pharmacy, Anhui Medical University, Hefei, Anhui, China; ^2^ Department of Pharmacy, North China University of Science and Technology, Tangshan, Hebei, China; ^3^ Hefei Kualai Biomedical Technology Co., Ltd., Hefei, Anhui, China; ^4^ The First Affiliated Hospital of USTC, Division of Life Science and Medicine, University of Science and Technology of China, Hefei, Anhui, China; ^5^ Guoke Saifu (Shenzhen) New Drug Research and Development Technology Co., Ltd., Shenzhen, China; ^6^ Hefei Anyaohui Health Industry Co., Ltd., Hefei, China; ^7^ State Key Laboratory of Drug Delivery Technology and Pharmacokinetics, Tianjin Institute of Pharmaceutical Research, Tianjin, China; ^8^ Research Unit for Drug Metabolism, Chinese Academy of Medical Sciences, Beijing, China

**Keywords:** rare disease, orphan drugs, new drug development, gene therapy, nucleic acid drugs, stem cell therapy

## Abstract

As the incidence of rare diseases increases each year, the total number of rare disease patients worldwide is nearly 400 million. Orphan medications are drugs used to treat rare diseases. Orphan drugs, however, are rare and patients often struggle to utilize them and expensive medications during treatment. Orphan drugs have been the focus of new drug research and development for both domestic and international pharmaceutical companies as a result of the substantial investment being made in the field of rare diseases. Clinical breakthroughs have been made in every field, from traditional antibodies and small molecule drugs to gene therapy, stem cell therapy and small nucleic acid drugs. We here review the therapeutic means of rare diseases and drug development of rare diseases to show the progress of treatment of rare diseases in order to provide a reference for clinical use and new drug development of rare diseases in China.

## Introduction

Rare diseases are a group of diseases that are uncommon and have a very low prevalence compared to other diseases (Haendel et al., 2020). The World Health Organization (WHO) defines diseases with a prevalence of 0.65‰–1‰ of the overall population as rare diseases (Schieppati et al., 2008). The latest definition of rare diseases in China is to meet at least one of the following three criteria: the incidence of newborns is less than 1/10,000, the prevalence rate is less than 1/10,000, and the affected population is less than 140,000 (Lu and Han, 2022). Rare diseases are rare among individuals, but their global prevalence is estimated at 3.5%–5.9%, affecting up to 400 million people. (Nguengang Wakap et al., 2020). To date, between6,000 and 8,000 rare diseases have been identified worldwide, and the number is increasing, with nearly 250 new diseases being classified as rare diseases each year (Hartin et al., 2020; Nguengang Wakap et al., 2020). According to statistics, there are about 20 million rare disease patients in China, and more than 200,000 new patients are increased every year, which shows that the number of patients affected by rare diseases is extremely large (Liu et al., 2019). There are 121 kinds of rare diseases in China’s First List of Rare Diseases, among which the common ones are epidermolysis bullosa (EB), albinism, ALS, spinal muscular atrophy (SMA), hemophilia (Dong, 2018). However, there are still many rare diseases that are not included in the Chinese rare disease catalog, such as Gorham-Stout, Cloves syndrome, autosomal recessive genetic disease and other rare diseases among rare diseases.

How to cure rare diseases and how to discover new drugs for rare diseases is a common problem facing the global pharmaceutical industry. Currently, only 10% of rare diseases in the world are treated accordingly, and more than 4,000 rare diseases are not cured accordingly (Sharma et al., 2010). Despite the low frequency of rare diseases, there is a high social need for cures and medication research for rare diseases in China, where there are over 1.4 billion people. Currently, there is a serious lack of treatment measures and treatment options for rare diseases, and many patients with rare diseases around the world are still not receiving timely and effective treatment. Consequently, in this review, we focus on analyzing and summarizing the current treatments and therapeutic drugs for rare diseases, including drugs that have been marketed, the latest clinical trials and hot drugs under investigation, with the aim of providing a reference for those involved in the study of rare diseases.

## Treatment methods for rare diseases

Accurate diagnosis and timely treatment of rare diseases are common challenges for clinicians. Genetic defects are a significant factor responsible for rare diseases. The treatments for hereditary rare diseases include diet therapy, surgery, medication, bone marrow transplantation, and et al. (Jiang et al., 2011). Small molecule medications, antibody therapy, enzyme replacement therapy, gene therapy, stem cell therapy, and drug repositioning are some of the modalities used to treat rare diseases. (Tambuyzer et al., 2019). In particular, the emergence of new research hotspots such as oligonucleotide therapy, stem cell therapy and gene therapy has altered the treatment pattern of many complicated diseases and brought fresh life to patients with rare diseases.

### Gene therapy

Gene therapy is an experimental technique to treat and prevent diseases by altering human genes (Gui et al., 2020). Adenoviral vector is one of the most commonly used viral vectors in gene therapy. It is capable of treating not only hematological diseases such as hemophilia, gaucher disease, and hemochromatosis, but also has made significant progress in treating genetic diseases, rare diseases, and tumors (Mingozzi and High, 2013; Gui et al., 2020). Inheritance is a critical factor in rare diseases (Dunbar et al., 2018). Most rare diseases are caused by mutations in a single gene, which can be inherited from parents or can occur spontaneously in germ cells (Rahit and Tarailo-Graovac, 2020). Most gene therapies are used for the treatment of rare disease. Glybera®, the first gene therapy drug approved by the European Medicines Agency in 2012, was developed by the Dutch company UniQure and is used to treat super rare disease lipoprotein lipase deficiency (Wirth and Yl-Herttuala, 2013). Since then, gene therapy based on DNA has led to several breakthroughs in the field of rare diseases. For an instance, the results of the Phase III clinical trial of Valoctocogene Roxaparvovec, an AAV gene therapy developed by BioMarin to treat adults with hemophilia major type A, were officially released (Long et al., 2022). Zynteglo, a Bluebird transfusion-dependent beta-thalassemia gene therapy, was approved in Europe (Schuessler-Lenz et al., 2020). In comparison to other countries, few independently developed gene therapies exist in China. The first gene therapy for type 1 SMA approved for clinical use in China is the AAV gene therapy EXG001-307 injection developed by Hangzhou Jiayin Biological Company (China company). Up to now, more than 40 gene therapy drugs have been listed in the world.These include *in vitro* gene therapy, virus-based *in vivo* gene therapy, and small nucleic acid drugs. Most gene therapies are used to treat rare diseases, such as in (Table 1). Additionally, several gene therapies are expected to be approved for marketing or submitted to BLA in 2022 based on publicly available information, among which the gene therapies that may gain progress in the field of rare diseases are shown in (Table 2). Gene therapy, as a new therapy that is expected to cure rare diseases at one time, has attracted international attention for a long time. The best time for the advancement of the gene therapy field is now, when it is still in its early stages of development and both basic research and societal support are urgently needed to be improved.

**TABLE 1:** Marketed genetic drugs in rare diseases.

^Disease^	^Therapy name^	^Name of the company^	^Target mechanism^	^Approval authority^	^Approval time^
^Lipoprotein deficiency hereditary disease^	^Glybera^	^UniQure^	^AAV-based gene therapy^	^EC^	^2017/10 (Delisting)^
^Spinal muscular atrophy^	^Zolgensma^	^AveXis (Novartis)^	^Gene therapy for expressing SMN protein^	^FDA^	^2019/5/24^
	^Spinraza^	^Ionis Pharmaceuticals^	^Gene therapy for expressing SMN protein^	^FDA^	^2016/12/23^
^Severe immunodeficiency^	^Strimvelis^	^Orchard Therapeutics^	^Gene therapy for expression of adenosine deaminase^	^EC^	^2016/5^
^Porphyria^	^Givlaari^	^Alnylam^	^Targeted ALAS1 gene therapy^	^FDA^	^2019/11^
^β-mediterranean anemia^	^LentiGlobin^	^Bluebird bio^	^Autologous stem cell therapy based on lentivirus^	^EC^	^2019/5^
	^Zynteglo^			^FDA^	^2022/8/17^
^Familial hypercholesterolemia^	^Leqvio^	^Novartis^	^Gene therapy targeting PCSK9^	^EC^	^2020/12/23^
	^Kynamro^	^IonisPharma/Kastle^	^Expression of apo B-100 messenger RNA as the target^	^FDA^	^2013/1/29^
^Adrenoleukodystrophy^	^Skysona^	^Bluebird bio^	^Gene therapy of ALD protein expression^	^EC^	^2021/7/21^
	^Libmeldy^	^Orchard Therapeutics^	^Gene therapy for expression of functional enzyme ARSA^	^EC^	^2022/1/10^
^Retinal nutritional atrophy^	^Luxturan^	^Spark Therapeutics^	^Retinal dystrophy associated with RPE65 mutation^	^FDA^	^2022/1/10^
^Mantle cell lymphoma^	^Tecartus^	^Kite Pharma^	^CD19 targeted CAR-T cell therapy^	^FDA^	^2020/7/24^
^Or relapsed or refractory multiple myeloma^	^Abecma^	^Bluebird bio/^	^Targeted b cell maturation antigen^	^FDA^	^2021/3/26^
^Aromatic L-amino acid decarboxylase^	^Upstaza^	^PTC Therapeutics^	^AAV5-based gene therapy^	^EC^	^2022/7/20^
^Hemophilia A^	^Roctavian^	^BioMarin^	^Gene therapy for expression of coagulation factor ⅷ^	^EC^	^2022/8/24^

Data sources are public information: https://www.fda.gov/, https://ec.europa.eu/info/index_en.

**TABLE 2:** List of gene therapies expected to gain ground in rare diseases in 2022.

Therapy name	Name of the company	Indication	Target mechanism	Expected time
Etranacogene dezaparvovec	UniQur and CSL Behring	Hemophilia B	Gene therapy for expression of coagulation factor ⅸ	In the second half of 2022
Lenadogene nolparvovec	GenSight Biologics	Leber hereditary optic neuropathy	Gene therapy for expressing NADH dehydrogenase 4	The fourth quarter of 2022
OTL-103	Ochard Therapeutics	Wiskott-Aldrich syndrome	Gene therapy for expressing WAS protein	Mid-2022
Elivaldogene autotemcel	bluebird bio	adrenoleukodystrophy	Gene therapy of ALD protein expression	Before September 2022
Beremagene geperpavec	Krystal Biotech	Dystrophic epidermolysis bullosa	Local gene therapy forexpression of collagen type VII (COL7)	In the second half of 2022
EB-101	Abeona Therapeutics	Dystrophic epidermolysis bullosa	Target therapy for expressing COL7A1 gene	Late 2022

Data sources are public information: https://www.fda.gov/, https://ec.europa.eu/info/index_en.

### Stem cell therapy

Hematopoietic stem cell transplantation (HSCT) is a treatment modality that uses human cells to modulate the immune system or to provide educational support (Alessandrini et al., 2019). There are several pathways of transplantation for HSCT, including fetal liver cell transplantation, bone marrow transplantation, peripheral hematopoietic stem cell transplantation, and umbilical cord blood transplantation. Many clinical practices and applications have demonstrated that cord blood has beneficial effects in treating rare diseases. (Islami and Soleimanifar, 2020). Previous study suggested that umbilical cord blood stem cells (UCBT) has significant efficacy in the treatment of primary rare diseases, but the slow immune reconstruction and high cytomegalovirus (CMV) infection rate are the defects that stem cell therapy can’t be ignored (Chen et al., 2017). Nevertheless, the prospects of umbilical cord blood in the treatment of rare diseases are good. For example, umbilical cord blood has brought the hope of rebirth to the rare diseases such as immunodeficiency syndrome, metachromatic leukodystrophy, chronic granulomatosis, IgE syndrome, etc., and has also achieved good therapeutic effects in a variety of lysosomal storage diseases (mucopolysaccharide storage enzyme, Gaucher’s disease, Niemann’s disease, etc.) (Chen et al., 2017).

Gaucher disease is the most common lysosomal storage disorder caused by glucocerebrosidase deficiency, and it is an autosomal recessive disorder (Bahnson et al., 1994). Current treatments for this disease include enzyme replacement therapy (ERT) and HSCT, a method that can provide a permanent source of enzymes to patients with Gaucher disease and is a cheaper procedure compared to the commonly used intervention ERT, which has been transplanted to treat Gaucher disease as early as 1984 (Somaraju and Tadepalli, 2017). There are numerous clinical cases of using cord blood to treat Gaucher disease in China. Some scholars reported three cases of non-hematopoietic cord blood stem cell transplantation for Gaucher’s disease (Tang et al., 2015). The PREVYMI oral and intravenous fluids from Merck, which help stop CMV infection following HSCT, received FDA approval in 2017. This is the first new drug approved by the FDA for CMV infection, and it promotes implantation while also strengthening infection prevention and control and improving HSCT success rates. HSCT is gradually being recognized and accepted by everyone, and it has a wide range of applications in rare diseases.

### Antibody therapy

Antibody therapy is a kind of passive immunity, that is, the human body passively accepts antibodies *in vitro*, thus obtaining specific immunity. For example, Muronomab-CD3 is the first therapeutic monoclonal antibody (mAb) approved for organ allograft rejection in 1986 (Buss et al., 2012). Antibodies play a role by regulating the signal transduction pathway, collecting cells or proteins to specific sites, and then delivering cytotoxic or neutralizing circulating factors (António et al., 2019). Cablivi (caplacizumab), the first nanobody drug that can be used to treat adult patients with acquired thrombotic thrombocytopenic purpura, was approved for marketing by the European Commission (EC) in 2018. Some academics (Chen et al., 2021) have provided suggestions and references for the research and development of clinical medications for rare diseases by summarizing the development of caplacizumab. Antibody therapies were developed primarily for indications with larger patient populations and then moved to some rare disease indications. For example, Alexion Pharm’s Soliris, a monoclonal antibody against the terminal complement protein C5, was first approved by the FDA in 2007 for the treatment of paroxysmal nocturnal hemoglobinuria (Kulagin Aleksandr et al., 2019). Moreover, Soliris is approved for the treatment of two other rare diseases that are closely related to the complement system: atypical hemolytic uremic syndrome and myasthenia gravis (Bernuy-Guevara et al., 2020). In summary, a major benefit of antibody treatments is its high specificity, which reduces the risk of off-target toxicity common in small molecule formulations and is crucial for the treatment of rare disease.

### Enzyme replacement therapy

Enzyme replacement therapy (ERT) can be used to treat partial lysosomal storage disease (LSD). The first time ERT was utilized to treat LSD was in 1980, when Brady (Brady et al., 1973) and colleagues created an enzyme replacement therapy for type 1 Gaucher illness. This served as proof-of-principle for the treatment of LSD that followed. ERT is a type of biologic therapy that was initially approved for Gaucher disease in 1991. Genzyme developed a recombinant form of glucocerebrosidase, which was first approved by the FDA in 1994 (Oprea et al., 2011). For the treatment of Gaucher disease, Fabray disease, Pompe disease, and mucopolysaccharide storage diseases, 15 enzyme replacement therapy rare illness medications have been approved for international marketing (Cheng et al., 2020). Late-onset Pompe disease is a rare disease. ERT has been approved for Pompe disease since 2006. It has been shown that ERT improves motor respiratory function in patients with late onset Pompe disease (Stockton et al., 2020). Nexviazyme, an enzyme replacement agent, was approved by the FDA in 2021 for the treatment of patients with (LOPD) aged 1 year and older (Xia, 2010). It can be seen that the focus of ERT development has always been on various LSDs, a genetic disease, causing enzyme deficiency or enzyme errors in lysosomes. Based on the ERT treatment of diseases for many years, ERT therapy has the advantages of better technical development and higher safety. However, there are also problems such as unclear distribution of biological drugs in the body, difficulty in crossing the blood-brain barrier, and expensive treatment costs (Brady et al., 1973). In order to successfully conduct clinical trials for ERTs, it is crucial to identify pertinent research endpoints and recognize the minimal clinically meaningful patient differences for these endpoints.

## Rare disease drug development

Just as rare diseases are called “ orphan diseases,” drugs to treat diseases are also called “ orphan drugs.” Drug treatments for rare diseases are in short supply. The catalog of “The First Batch of Rare Diseases” released by China covers 121 rare diseases, of which 105 diseases have no targeted drug treatment yet, accounting for 87% of the 121 rare diseases, yet 51.85% of the 27 orphan drugs listed in China are completely dependent on imports (Liu et al., 2019). There is still a gap between the current situation of rare disease drugs in China and international developed countries and regions, and there is a serious shortage of rare disease drugs and insufficient reserve of new drug research and development (Ji et al., 2019). The reason for this is that a new drug needs to go through a long process and huge capital investment from laboratory research and development to end product launch, and the number of patient groups corresponding to rare disease drugs is relatively small. This makes it difficult for profit-seeking pharmaceutical companies to judge revenues and, therefore, to be discouraged from developing and producing generic products.

The study of the human genome and developments in molecular biology have opened up new avenues for the discovery of novel medicines. From the initial therapeutic agents based on protein (protein, peptide and antibody) to the recent nucleic acid therapy, gene and cell therapy, the focus of new drug research and development is no longer limited to antibodies, protein and traditional small molecule drugs, but small nucleic acid drugs, stem cell drugs and enzyme protein degradation agents based on PROTAC technology are the key directions of new drug research and development.

### Small molecule drugs

The research and development of small molecule drugs have always been the focus of pharmaceutical companies and is in the mainstream of drug discovery and development. Computer-aided screening and technological advances in chemistry and biology have improved the timeliness of discovering and designing new drugs, especially in the field of small molecules, and artificial intelligence (AI)-assisted research and development of new drugs has become hot (Tripathi et al., 2021). It can use the existing data to analyze and integrate the literature information in the database, and can quickly identify and screen the compounds that are effective for the treatment of rare diseases. For example, the Benevolent Bio (UK company) used the technology platform JACS to screen out 5 compounds from 100 potential compounds that can treat ALS and confirmed that 4 of them were effective in curing motor neurodegeneration (Liu, 2018). National support for innovative medicines continues to grow and AI is driving a new wave of small molecular development (Schmidt and Thompson, 2020). The success of small molecule drugs in rare diseases has been driven by targeted screening and better-quality disease models, by introducing more efficient screening techniques and developing chemical libraries for filtering to exclude undesirable drug structures. For example, for the treatment of cystic fibrosis, Vertex first developed the CFTR enhancer ivacaftor based on *in vitro* screening, then a diphasic formulation of lumacaftor and ivacaftor that facilitates CFTR transport was developed under the trade name Orkambi for the treatment of F508del mutant CF, and finally the company developed a triple formulation of Trikafta targeting one or both F508del alleles (Zaher, 2021). LSDs are fairly common examples of small compounds that have the potential to target all organs. In response to the shortcomings of ERT preparation, which is costly and requires injection and cannot penetrate the central nervous system (CNS), two small molecule therapies for LSDs that inhibit the biosynthesis of defective enzyme substrates, Zavesca from Actelion Pharms and Cerdelga from Genzyme have been approved for the treatment of Gaucher disease in LSDs. In conclusion, AI technologies using deep learning methodologies and novel techniques for phenotypic screening to locate compounds with the required therapeutic potential can uncover new indications for medicines, which may potentially be useful for uncommon diseases with unknown etiologies.

### Small nucleic acid drugs

Small nucleic acid drugs are developing rapidly in the field of biopharmaceuticals, which consist of nucleotides with specific designed sequences. Due to the breakthroughs in nucleic acid modification and delivery vector technologies in recent years, small nucleic acid drugs are now one of the effective tools for the treatment of rare diseases (Barkau et al., 2021). Small nucleic acid drugs mainly include nucleic acid drugs such as antisense oligonucleotides (ASO), small interfering RNA (siRNA), microRNA (miRNA) (Yamada, 2021). Because of both genetic modifications and traditional drugs, breakthroughs have also been made in the treatment of genetically inherited diseases. For instance, Nusinersen, the first medication ever to be licensed by the FDA to treat SMA, works by attaching to the precursor mRNA created by the transcription of the SMN2 gene, changing the RNA splicing pattern, and subsequently enhancing the expression of normal SMN proteins (Finkel et al., 2021). Milasen is an antisense oligonucleotide containing 22 nucleotides with the same backbone and glyco-chemical modifications as Nusinersen, which can be used to treat Batten disease (Mitchell et al., 2018). There are currently 14 small nucleic acid drugs marketed worldwide, including 9 ASO drugs, 4 siRNA drugs, and 1 nucleic acid aptamer (Table 3).

**TABLE 3:** List of global marketed small nucleic acid drugs.

Category	Pharmaceuticals	Enterprise	Indications	Time to market
siRNA	Onpattro	Alynlam	Familial amyloid polyneuropathy	2018
siRNA	Givlaari	Alynlam	Acute hepatic porphyria	2019
siRNA	OXLUMO	Alynlam	Primary hyperoxaluria type1	2020
siRNA	Inclisiran	Alynlam, Novartis	Adult hypercholesterolemia and mixed dyslipidemia	2020
ASO	Vitravene	lonis, Novartis	Cytomegalovirus retinitis	1998
ASO	Kynamro	lonis, Sanofi	Familial hypercholesterolemia of the pure type	2013
ASO	Exondys51	Sarepta Therapeutics	Duchenne muscular dystrophy	2016
ASO	Spinraza	Lonis, Biogen	Spinal muscular atrophy	2016
ASO	Tegsedi	lonis	Hereditary amyloidosis of transthyretin protein	2018
ASO	Waylivra	lonis	Familial celiac disease syndrome	2019
ASO	Vyondy53	Sarepta Therapeutics	Duchenne muscular dystrophy	2019
ASO	Viltepso	Nippon Shinyaku	Duchenne muscular dystrophy	2020
ASO	AMONDYS45	Sarepta Therapeutics	Duchenne muscular dystrophy	2021
Aptamer	Macugen	Eyetech, Pizer	Neovascular age-related macular degeneration	2004

Data source: https://www.cn-healthcare.com/articlewm/20210410/content-1208704.html.

Eighty percent of these are antisense oligonucleotide acid drugs used for the treatment of rare genetic disorders, particularly for Duchenne muscular dystrophy. Duchenne muscular dystrophy (DMD), also known as pseudohypertrophic muscular dystrophy, is the most symptomatic form of muscular dystrophy (Brunetti et al., 2020). Eteplirsen, the first FDA-approved gene therapy for DMD, is an antisense nucleotide drug specifically designed for exon 51 (Rowel et al., 2017). In addition to Eteplirsen, there are three other products targeting different genetic loci: Golodirsen in 2019, Viltolarsen in 2020 (DMDE53), and Casimersen in 2021 (DMDE45) (Li et al., 2020; Hu and Dong, 2021; Zhang and Hu, 2022). Small nucleic acid medications are regarded as a new class of drug development following small molecule drugs and protein drugs because they have numerous advantages over small molecule drugs, including the ability to target many disease proteins that are untargetable. This gives the treatment of rare diseases new hope.

### Hematopoietic stem cell drugs

One of the important values of stem cells is reflected in their application in disease treatment, especially in the treatment of rare diseases has also made some progress on the road, and some rare diseases have been treated internationally with corresponding therapeutic drugs. For example, proximal developed by Osiris (United States company) was approved by FDA for marketing, which is the first stem cell therapy drug for Crohn’s disease and childhood graft-versus-host disease (Patel and Genovese, 2011). In 2012, the FDA approved the marketing of MultiStem, a stem cell therapy by Athersys (United States company), for the indication of type I mucopolysaccharide storage disease (Robert and Deans, 2016). Combining stem cell therapy with gene therapy, 4 hematopoietic stem cell gene therapies have been launched in Europe, 3 of which are used for rare diseases. Adenosine deaminase deficiency causes severe combined immunodeficiency illness, which is treated with strimvelis®, a gene therapy authorized in 2016 (South et al., 2019). Libmeldy, approved by Orchard Therapeutics in 2020, was launched for the treatment of early-onset heterozygous cerebral leukodystrophy. Skysona, which Bluebird Bio authorized in 2021, is used to treat adrenal cerebral leukodystrophy (ProQuest, 2021). Some of the indications for stem cell therapies currently in development are being extended to rare diseases, such as Temcell, a bone marrow mesenchymal stem cell product for graft-versus-host disease that was launched in Japan in 2016 and is being expanded this year into studies for the treatment of Epidermolysis bullosa (Wu, 2022). Additionally, stem cell treatments for uncommon illnesses like Parkinson’s and acromegaly are all entering late stages of clinical trials and are getting closer to going on sale.

### Enzymatic protein degraders based on Protein degradation targeting chimera technology

Protein degradation targeting chimera (PROTAC), after more than 20 years of improvement and development, is one of the hot spots in the field of new drug research and development, providing a brand-new direction for enterprises to develop new drugs. Compared with traditional small molecule drugs, it is able to modulate many targets that cannot be reached by small molecules or antibodies. The core of this technology is to design small molecule compounds into novel drugs and solve the drug resistance problem that often occurs with small molecule inhibitors by degrading the target protein (Paiva and Crews, 2019). Haisco Pharmaceutical Group Co., Ltd. is the first company in China to submit a clinical filing for PROTAC, and its KSK29116 can be used to treat relapsed refractory B-cell lymphoma. Combining the benefits of small molecule drugs and small molecule nucleic acids, PROTAC has a very broad application and development potential in the field of rare diseases. Additionally, its method of controlling the protein content *in vivo* by degrading target proteins greatly expands the potential. The range of potential therapeutic targets has been substantially increased by the method of regulating the protein composition *in vivo* by degrading target proteins (Li et al., 2022).

## Prospects of treatments for rare diseases

### Application of gene therapy in ophthalmology

Currently, rare diseases are primarily dependent on genetic diagnostics and pharmacotherapy. Since 2022, AAV gene therapy has continued to play a role in the area of neuromuscular rare diseases, showing great potential for development in the division of rare ocular diseases.

Age-related macular degeneration (AMD), a disease capable of causing central vision loss and retinal lesions (Mercuri et al., 2018). Molecular Therapeutics reported on 6 January 2022, that 4D-150, an intravitreal transgenic AAV gene therapy, was given to the first patient in a mid-term clinical trial. REGENXBIO announced interim data from the Phase 2 ALTITUDE trial of its ophthalmic AAV gene therapy RGX-314 in February 2022. The clinical trial application for “KH631 Ophthalmic Injection” filed by Chengdu Hongji Biotechnology Co., Ltd. (China company). All three of these drugs are based on AAV gene therapy for the treatment of wet age-related macular degeneration (wAMD).

As there is no clinically effective treatment for Leber’s hereditary optic neuropathy (LHON), gene therapy is currently the most promising and effective treatment for LHON (Chen et al., 2017). LUMEVOQ, an AAV gene therapy for Leber’s hereditary optic neuropathy, reported long-term clinical data from GenSight Biologics in January 2022, which showed that subjects continued to experience significant improvements in vision four years after a single gene therapy injection. In a mid-stage clinical trial to treat pigmentary retinitis brought on by mutations in the NR2E3 and RHO genes, Ocugen’s AAV gene therapy OCU400 completed its first patient dosage in April 2022. According to the center for drug evaluation (CDE), on 19 April 2022, Shanghai Langsheng Biotechnology Co., Ltd. (China company) received a clinical trial implied license for a new class 1 drug, LX101 injection, for the treatment of hereditary retinal degeneration associated with a double allele mutation in RPE65. Applied Genetic Technologies Corporation (AGTC) announced positive results from its ongoing mid-stage study of the AAV gene therapy AGTC-501 for the treatment of X-linked retinitis pigmentosa (XLRP) on 4 May 2022. It has been demonstrated that the persistence of improving visual function exceeds that of 18 yuan for months, and provides biological activity evidence for XLRP gene therapy.

### Application of combined therapy in rare diseases

Because most gene therapy uses AAV as a carrier to deliver therapeutic genetic materials, it makes patients naturally immune to these viruses, which leads to ineffective treatment. To solve this problem, the emerging exosomes technology provides an effective treatment for it. The use of exosomes to deliver gene drugs in cells will not induce adaptive immune response, so more and more companies are starting to cooperate with platforms using exosome technology. Sarepta, for instance, has succeeded in cooperating with Codiak to jointly design and develop gene therapy based on exosome engineering technology. The former is a company that develops gene therapy for rare diseases and a leader in the field of precise gene therapy for rare diseases, while the latter has a special exosomes technology platform. The benefits of exosomes technology have also drawn many investors. In short, the discovery of exosomes has brought a great leap in the field of gene therapy, and exosomes-mediated therapy will definitely make great progress in the field of gene delivery in the future. In addition to gene therapy, stem cell therapy is also a new focal point for rare disease research. Stem cells are making strides in the treatment of rare diseases. Some rare diseases in the world already have corresponding stem cell therapy drugs. With the development of its technology, some scholars believe that the most effective stem cell therapy in the future may be on rare diseases. In addition, the combination of gene technology and stem cell technology can make more rare diseases end the dilemma of no medicine, such as treating EB with genetically modified stem cells.

### Suggestions on speeding up research and development of rare disease drugs

Nowadays, under the influence of Corona Virus Disease 2019 (COVID-19), our government funds are facing great pressure, the funds to support rare diseases are limited. There is a small domestic market and fierce competition between innovative companies. The current conditions bring difficulties to the research and development of rare drugs. However, even under many difficulties, enterprises and personnel involved in this field should keep confidence and hope. Domestic businesses can take a different approach and focus on research and development of rare diseases that have never been seen abroad with low incidence, despite the fact that they are somewhat behind overseas businesses in this regard. Government departments should further establish a sound medical insurance system for rare diseases and improve national health insurance policies. Companies are expected to increase their innovation and imitation efforts and actively introduce relevant innovative talent. Prevention is the most important factor in the diagnosis and treatment of rare diseases. Important measures to prevent rare diseases include avoiding consanguineous marriages, avoiding births at advanced ages, genetic counseling and prenatal diagnosis. In addition, publicity and scientific knowledge about rare diseases should be carried out. In summary, there is an urgent need to improve our capacity to treat rare diseases and develop new drugs.

## Conclusion

The development of new pharmaceuticals is focusing on gene therapies, stem cell therapies, and small nucleic acid therapies, which is altering the therapy landscape for many diseases that are challenging to treat. For example, there are therapeutic effects in blood diseases, oncological diseases, and immune system diseases. Increasing evidence have showed that these therapies also play a critical role in the treatment of rare diseases. In summary, significant advances in the treatment of rare diseases are expected in the future.

## Abbreviations

ALS, amyotrophic lateral sclerosis; SMA, spinal muscular atrophy; WHO, World Health Organization; AAV, adeno-associated virus; HSCT, hematopoietic stem cell transplantation; UCBT, umbilical cord blood transplantation; CMV, cytomegalovirus; ERT, enzyme replacement therapy; EC, European Commission; EB, epidermolysis bullosa; LSD, lysosomal storage disease; CNS, central nervous system; DMD, Duchenne muscular dystrophy; AMD, Age-related macular degeneration; LHON, Leber’s hereditary optic neuropathy; AGTC, Applied Genetic Technologies Corporation; XLRP, X-linked retinitis pigmentosa.
